# Management of Uncomplicated Plasmodium vivax Malaria in a Pregnant Patient With G6PD Deficiency

**DOI:** 10.7759/cureus.110364

**Published:** 2026-06-06

**Authors:** Richa Y Patel, Jessica M Ngo, Aashna N Maknojiya, Armando Meza

**Affiliations:** 1 Infectious Diseases, Texas Tech University Health Sciences Center, El Paso, USA

**Keywords:** antimalarial drugs, drug-induced hemolytic anemia, first-trimester of pregnancy, glucose-6-phosphate dehydrogenase (g6pd) deficiency, plasmodium vivax malaria, primaquine contraindication, us-mexico border, vector borne disease

## Abstract

*Plasmodium vivax* is one of the most common forms of malaria worldwide, notable for its ability to relapse from a dormant liver stage. Radical cure of *Plasmodium* as well as with* P. ovale* requires the use of 8-aminoquinolines to clear both blood and liver parasites; however, they are contraindicated in glucose-6-phosphate dehydrogenase (G6PD) deficiency due to the risk of causing hemolysis and in pregnant individuals due to the unknown G6PD status of the fetus.

We present a case of a young pregnant woman with fever, nausea, and vomiting, who, upon blood peripheral smear and polymerase chain reaction (PCR) confirmation, was diagnosed with *Plasmodium vivax*. In this case, standard radical cure was not feasible during pregnancy, and a seven-day course of quinine and clindamycin was initiated for treatment of the acute blood-stage infection while G6PD results were awaited. Upon discharge, the patient was unable to obtain the medication and returned two months later with relapsing fevers, abdominal pain, and chills.

Given presumed chloroquine-sensitive *Plasmodium vivax*, chloroquine remains the preferred agent for suppressive therapy during pregnancy. However, hydroxychloroquine was subsequently selected due to its more favorable side effect profile and improved tolerability for prolonged use. Both agents are considered safe in pregnancy.

Before initiating treatment, G6PD screening is essential, as many antimalarial agents can precipitate hemolysis; quantitative testing is particularly important to distinguish deficiency from intermediate activity in heterozygous females.

This case underscores the complexities of managing *P. vivax* infection in overlapping contraindications. It highlights the critical role of early and precise G6PD assessment, with quantitative assays in females being particularly essential, to inform safe administration of antimalarial therapy. These findings emphasize the necessity of individualized management strategies, especially for vulnerable migrant populations, and the importance of a structured follow-up plan to prevent relapse and ensure the delivery of definitive treatment.

## Introduction

Malaria is a global health concern with a significant disease burden, responsible for about 263 million cases in 2023 [1]. Malaria is caused by infection from a single-celled protozoan parasite of the *Plasmodium* genus that is spread by bites from an infected *Anopheles *mosquito [2].

Five *Plasmodium* species are known to infect humans: *P. falciparum, P. vivax, P. ovale, P. malariae, *and* P. knowlesi, *each causing disease with characteristic clinical presentations that vary in severity [3]. The most common is *P. vivax*, the species our patient presented with. Along with *P. ovale*, it is known for its ability to form dormant liver parasites called the hypnozoite phase. These hypnozoites are especially dangerous because they can remain in the liver and reactivate weeks or months after the initial infection [1]. If not treated initially to remove the parasite from the liver, patients can relapse with the symptoms of initial infection; thus, this requires treatment with 8-aminoquinoline medications such as primaquine or tafenoquine, a drug commonly used as prophylaxis, targeting the dormant liver stage as well as the active blood parasites [4]. 

However, treatment is complicated in patients with glucose-6-phosphate dehydrogenase (G6PD) deficiency, an X-linked enzyme disorder predisposing red blood cells to be more vulnerable to oxidative stress. Without sufficient G6PD, the body cannot regenerate glutathione, an important antioxidant, leading to hemolysis under oxidative conditions such as infection or treatment with certain medications, such as the 8-aminoquinolines mentioned previously [5].

Furthermore, pregnancy introduces another challenge to the case as 8-aminoquinolines are contraindicated due to the unknown status of whether the fetus is also G6PD deficient and the potential risk of hemolytic anemia [6].

This case presents a unique clinical case, as the patient tested positive for *P. vivax* and was found to have G6PD deficiency while also being in the first trimester of pregnancy. These co-existing conditions complicate treatment, as the most effective antimalarials for relapse prevention are contraindicated in both pregnancy and G6PD deficiency.

Additionally, the patient is a recent migrant, and it remains unclear whether she previously received treatment for *P. vivax* or was unable to complete it, raising concerns about possible drug resistance. With few comparable cases in the literature, this scenario highlights the urgent need for clear treatment guidelines for patients with overlapping contraindications. It also emphasizes the importance of standardized G6PD testing, particularly quantitative methods, to guide safe and effective management in high-risk populations.

The abstract of this article was previously presented at the American Federation for Medical Research (AFMR)/Western Society for Pediatric Research (WSPR) 2026 Western Medical Research Conference, held January 15-17, 2026, in California, USA.

## Case presentation

A 22-year-old pregnant woman in her first trimester presented to the emergency department with three days of nausea, vomiting, and new-onset fever. She had recently migrated from Venezuela and had arrived in the United States one week prior. Hematologic values found upon admission are presented in Table 1.

**Table 1:** Patient’s hematologic results upon admission Results are notable for mild anemia and thrombocytopenia, consistent with G6PD deficiency.

Parameter	Value	Reference Range
White Blood Cells (WBC)	7.26	4.5-11.0 x10^3^/µL
Red Blood Cells (RBC)	4.14	4.1-5.1 x 10^6^/µL (women)
Hemoglobin (Hb)	11.9	12-16 g/dL (women)
Hematocrit (Hct)	34.7	36%-45% (women)
Platelet Count	129	150-450 × 10³/µL
Mean Platelet Volume (MPV)	10.6	7.5-12 fL
Sodium	130	136-142 mmol/L
Potassium	3.5	3.5-5.0 mmol/L
Blood Urea Nitrogen (BUN)	3	8-23 mg/dL (adults)
Creatinine	0.4	0.6-1.2 mg/dL (adults)
Albumin	3.4	3.5-5 g/dL (adults)
Total Bilirubin	1.2	0.3-1.2 mg/dL
Glomerular Filtration Rate (GFR)	143	90-120 ml/min/1.73 m²
G6PD	4.6	7.0-20.5 U/g Hb
G6PD Enzyme Activity Test	3.2	8.0-11.9 U/g Hb

Before receiving the G6PD results, the patient was empirically treated with quinine and clindamycin for seven days for acute blood-stage infection while testing was pending. Although quinine is typically combined with a tetracycline for malaria treatment, tetracyclines are contraindicated in pregnancy due to risks of fetal bone and dental toxicity; therefore, clindamycin was used as a pregnancy-safe alternative [1]. Upon discharge, the patient was unable to obtain the prescribed medication and did not attend outpatient infectious disease follow-up. Kinetic spectrophotometry G6PD quantitative testing later revealed reduced enzyme activity at 4.6 U/g Hb. 

Approximately two months later, the patient, at 17 weeks’ gestation, returned to the emergency department with relapsing fevers, abdominal pain, and chills after being unable to obtain the prescribed therapy. Peripheral smear remained positive for intraerythrocytic parasites, and polymerase chain reaction (PCR) confirmed *Plasmodium vivax* infection. Repeat G6PD testing demonstrated enzyme activity of 3.2 U/g Hb (reference range: 8.0-11.9 U/g Hb), consistent with G6PD deficiency. The reported value of 32% activity was derived from a quantitative assay performed at the Mayo Clinic Laboratory, with “mean normal” defined according to the laboratory’s internal reference standard. Genotyping was considered to further characterize the variant; however, it was not performed in this case due to clinical constraints and management decisions based on phenotypic enzyme activity.

Given the inability to administer radical cure during pregnancy and the risk of relapse from persistent hepatic hypnozoites, suppressive prophylaxis was required. Weekly hydroxychloroquine was initiated until delivery. Although chloroquine is the preferred agent for suppressive prophylaxis in chloroquine-sensitive *P. vivax*, hydroxychloroquine may be used as an alternative when improved tolerability is desired. Mefloquine, which is safe in pregnancy and effective against chloroquine-resistant *P. vivax*, was considered but not selected due to its higher risk of neuropsychiatric adverse effects and concerns regarding long-term tolerability [1].

The patient returned for delivery at 37 weeks and five days and reported adherence to the weekly regimen. Following delivery, she was lost to follow-up after relocating out of town.

## Discussion

In non-pregnant cases of *P. vivax*, the recommended first-line treatment is chloroquine to treat the blood stage infection. Because *P. vivax* can form dormant hypnozoites in the liver, patients are at risk of relapse even if blood-stage parasites are eradicated. Treatment with chloroquine is then followed by primaquine or tafenoquine due to its ability to eliminate parasites in the blood and treat the liver stage of infection [6,7]. Primaquine and tafenoquine are 8-aminoquinolines, which are prodrugs metabolized by CYP2D6, a member of the cytochrome P450 family, and cytochrome P450 NADPH: oxidoreductase into active metabolites that generate hydrogen peroxide. This results in oxidative stress that is lethal to malarial parasites, but can also harm human cells [5]. Use of 8-aminoquinolines (primaquine, tafenoquine) is contraindicated in patients with G6PD deficiency due to the risk of inducing hemolytic anemia. Thus, G6PD status should be determined using appropriate methods to guide the safe administration of such drugs [1].

The testing of G6PD can be conducted through qualitative or quantitative measures. If G6PD status is unknown, quantitative testing is recommended to confirm normal G6PD activity, as qualitative tests may not detect individuals with intermediate deficiency [8]. Quantitative testing provides thresholds to determine if an individual has any deficiencies in G6PD activity. In males and females, if the G6PD activity is <30% of normal, the individual is considered deficient. In females, if the G6PD activity is between 30%-70% of normal, they can be categorized as having a heterozygous phenotype and an intermediate G6PD activity [1]. 

The three most common testing methods used today are the fluorescent spot test, spectrophotometric assay, and cytochemical assay. A key principle these tests utilize is the role G6PD plays in converting NADP+ to NADPH. In the fluorescent spot test, if an individual is deficient in G6PD, the blood will not fluoresce. This test is reliable for hemizygous males and homozygous females. It is not recommended to diagnose heterozygous females, as these individuals still produce enough NADPH to cause fluorescence, which could lead to a false-negative result [9,10]. Another frequently used test, the spectrophotometric assay, utilizes a spectrophotometer to measure the NADPH produced and is better at detecting hemizygous males and homozygous females. However, since this is a quantitative test, we can more accurately categorize an individual's G6PD status using the threshold values identified. Lastly, the cytochemical assay in which individual erythrocytes are stained if G6PD activity is present, and the conversion of exogenous glucose-6-phosphate and NADP+ occurs [9]. The percentage of stained and unstained erythrocytes can be manually determined through light microscopy; however, new methods utilizing flow cytofluorometric analysis can allow for easier detection [9,11]. Because this method allows for an objective analysis of stained and unstained erythrocytes, it can accurately detect hemizygously, homozygously, and heterozygously G6PD-deficient patients [9]. Table 2 summarizes the specificity and sensitivity of the three tests discussed.

**Table 2:** Specificity and sensitivity for each test type in detecting hemizygous, homozygous, and heterozygous G6PD-deficient individuals as stated in the literature Fluorescent spot tests and spectrophotometric assays have high specificity and sensitivity in hemizygous and homozygous individuals, while cytochemical assays have a higher specificity and sensitivity for heterozygous individuals [9,12,13].

	Specificity %		Sensitivity %	
	Hemizygous/Homozygous	Heterozygous	Hemizygous/Homozygous	Heterozygous
Fluorescent Spot Test	100	99	99	32
Spectrophotometric Assay	100	99	99	11
Cytochemical Assay	Not determined	85	Not determined	100

When screening a patient for G6PD deficiency before starting potentially hemolytic agents in the treatment of malaria, it is essential to consider an inexpensive and uncomplicated test that also accurately diagnoses a heterozygous female. All of the tests discussed above are relatively inexpensive, but differ in their ability to detect G6PD activity levels reliably. It has been recommended that two different tests be used for males and females. For males, a fluorescent spot test is sufficient to detect G6PD deficiency, but for females, a cytochemical assay should be used to differentiate between homozygous and heterozygous genotypes [9].

Additionally, 8-aminoquinolines are contraindicated in pregnancy due to the unknown G6PD status of the fetus and potential for severe hemolytic anemia. They are also contraindicated in breastfeeding mothers if the infant is G6PD deficient or if G6PD status is unknown due to the risk of exposure to 8-aminoquinolines through breast milk [6,8]. In pregnant patients, treatment with 8-aminoquinolines should be deferred until delivery, after which subsequent treatment is dependent on the mother’s G6PD status and whether she is breastfeeding. For women who are breastfeeding, 8-aminoquinolines can safely be prescribed if the infant is found to have normal G6PD activity. Women who are unable to take 8-aminoquinolines after delivery should be maintained on weekly chloroquine chemoprophylaxis for a total of one year after the acute malaria episode to prevent relapse [14].

The importance of this case is that it highlights the difficulties in treating *P. vivax* infections that coexist in a pregnant individual with a G6PD deficiency. *P. vivax* and *P. ovale* are both uniquely known to relapse through their latent hypnozoite forms, thus demanding a radical cure using 8-aminoquinolines, such as primaquine or tafenoquine. However, these are contraindicated in patients who are pregnant due to potentially causing fetal hemolytic anemia and the unknown status of G6PD in the fetus [6]. They’re also contraindicated in G6PD-deficient individuals due to the risk of hemolytic anemia, and in these cases, dose adjustment is necessary. 

Although artemisinin-based combination therapies (ACTs) are effective against blood-stage parasites, they too are not universally approved for use in the first trimester of pregnancy. 

In this case, quinine and clindamycin were selected as an appropriate empiric regimen given their safety profile in early pregnancy. Suppressive therapy with weekly hydroxychloroquine was employed as a temporizing measure to reduce the risk of relapse until definitive postpartum treatment could be administered. 

Because the patient could not recall whether she had previously been treated for malaria while residing in Venezuela, there was concern for possible prior exposure and potential drug resistance. Resistance has been reported to multiple antimalarial agents used for *Plasmodium vivax*, including chloroquine, the first-line therapy [15]. 

After delivery of the child, artemether-lumefantrine was recommended as definitive therapy for presumed chloroquine-resistant *Plasmodium* infection; however, the medication was not available at discharge, and the patient was subsequently lost to follow-up [16]. It is therefore unknown whether treatment was ever started or completed. Although artemether-lumefantrine is an accepted option for chloroquine-resistant malaria, its effectiveness in *P. vivax* is less consistent, and resistance patterns can vary. This introduces uncertainty regarding whether the selected therapy would have provided adequate coverage in this case, particularly given the unknown resistance status [1].

Definitive management of *P. vivax* also includes anti-hypnozoite therapy with low-dose primaquine to prevent relapse; however, this can only be initiated postpartum once breastfeeding considerations are addressed and the infant is confirmed to be older than one month and G6PD sufficient, or when breastfeeding has ceased [1]. These constraints highlight the importance of clearly defined post-delivery treatment pathways in patients with G6PD deficiency.

The need for more straightforward clinical guidelines for managing patients with overlapping contraindications calls attention to vulnerable populations, such as migrants, who may face barriers to continuous care.

## Conclusions

This case highlights the complex treatment challenges that arise when *P. vivax* malaria occurs in a patient who is both pregnant and G6PD-deficient. Due to the inability to complete the required treatment in this case, suppressive therapy is a required transition until postpartum definitive treatment is feasible. Clinicians are urged to proactively test for G6PD deficiency and select the appropriate type of testing, i.e., quantitative enzyme activity, to inform safe administration of antimalarial drugs like primaquine postpartum. Individualized, multidisciplinary treatment and follow-up over the long term are necessary to maximize outcomes and prevent relapse for this high-risk population.
